# Lactate-Based Difference as a Determinant of Outcomes following Surgery for Type A Acute Aortic Dissection: A Multi-Centre Study

**DOI:** 10.3390/jcm12196177

**Published:** 2023-09-25

**Authors:** Francesco Nappi, Almothana Alzamil, Antonio Salsano, Sanjeet Singh Avtaar Singh, Ivancarmine Gambardella, Francesco Santini, Antonio Fiore, Giacomo Perocchio, Pierre Demondion, Patrick Mesnildrey, Thibaut Schoell, Nicolas Bonnet, Pascal Leprince

**Affiliations:** 1 Department of Cardiac Surgery, Centre Cardiologique du Nord, 93200 Saint-Denis, France; almothana.md@gmail.com (A.A.); dr.mesnildrey@gmail.com (P.M.); tiboschoell@hotmail.com (T.S.); drnicolasbonnet@gmail.com (N.B.); 2 Division of Cardiac Surgery, Ospedale Policlinico San Martino, Italy DISC Department, University of Genoa, 16145 Genoa, Italy; ant.salsano@gmail.com (A.S.); francesco.santini@unige.it (F.S.); giacomo.perocchio@gmail.com (G.P.); 3 Department of Cardiothoracic Surgery, Weill Cornell Medicine–New York, Presbyterian Medical Center, 505 E 70th St., New York, NY 10065, USA; sanjeetsinghtoor@gmail.com; 4 Department of Cardiothoracic Surgery, Royal Infirmary of Edinburgh, Edinburgh EH16 4SA, UK; icg9002@med.cornell.edu; 5 Department of Cardiac Surgery, Hôpitaux Universitaires Henri Mondor, Assistance Publique-Hôpitaux de Paris, 94000 Créteil, France; fioreant7@yahoo.com; 6 Department of Cardiothoracic Surgery, Hôpital Pitié-Salpêtrière, Boulevard de Hôpital 47–83, 75013 Paris, France; demondion.pierre@hotmail.fr (P.D.); pascal.leprince@aphp.fr (P.L.)

**Keywords:** type A acute aortic dissection, arterial lactate, aortic root replacement, aortic arch repair, valve-sparing aorta replacement, ascending aorta replacement, total arch replacement procedure

## Abstract

Type A acute aortic dissection (TAAAD) is a serious condition within the acute aortic syndromes that demands immediate treatment. Despite advancements in diagnostic and referral pathways, the survival rate post-surgery currently sits at almost 20%. Our objective was to pinpoint clinical indicators for mortality and morbidity, particularly raised arterial lactate as a key factor for negative outcomes. Methods: All patients referred to the three cardiovascular centres between January 2005 and December 2022 were included in the study. The inclusion criteria required the presence of a lesion involving the ascending aorta, symptoms within 7 days of surgery, and referral for primary surgical repair of TAAAD based on recommendations, with consideration for other concomitant major cardiac surgical procedures needed during TAAAD and retrograde extension of TAAAD. We conducted an analysis of both continuous and categorical variables and utilised predictive mean matching to fill in missing numeric features. For missing binary variables, we used logistic regression to impute values. We specifically targeted early postoperative mortality and employed LASSO regression to minimise potential collinearity of over-fitting variables and variables measured from the same patient. Results: A total of 633 patients were recruited for the study, out of which 449 patients had complete preoperative arterial lactate data. The average age of the patients was 64 years, and 304 patients were male (67.6%). The crude early postoperative mortality rate was 24.5% (110 out of 449 patients). The mortality rate did not show any significant difference when comparing conservative and extensive surgeries. However, malperfusion had a significant impact on mortality [48/131 (36.6%) vs. 62/318 (19.5%), *p* < 0.001]. Preoperative arterial lactates were significantly elevated in patients with malperfusion. The optimal prognostic threshold of arterial lactate for predicting early postoperative mortality in our cohort was ≥2.6 mmol/L. Conclusion: The arterial lactate concentration in patients referred for TAAAD is an independent factor for both operative mortality and postoperative complications. In addition to mortality, patients with an upper arterial lactate cut-off of ≥2.6 mmol/L face significant risks of VA ECMO and the need for dialysis within the first 48 h after surgery. To improve recognition and facilitate rapid transfer and surgical treatment protocol, more diligent efforts are required in the management of malperfusion in TAAAD.

## 1. Introduction

In the contemporary age, type A acute aortic dissection (TAAAD) persists as a clinicopathological entity that poses a life-threatening risk coupled with a substantial possibility of mortality and morbidity. Fifty percent of patients with TAAAD die within 24 h. Estimates suggest that an additional 50% of patients may die before reaching a specialist aortic surgery referral centre [1,2,3,4,5,6,7,8]. The most effective treatment option for such patients is timely surgical repair. Thanks to greater clinical awareness, diagnostic techniques, and inceptive management [9], there has been a rise in the number of cases diagnosed promptly and undergoing TAAAD repair in the previous decade. However, despite reports of lower operative mortality rates not exceeding 7% [10,11,12,13], survival rates following surgical procedures remain suboptimal, with high rates of in-hospital mortality (16–18%) [1,2,3,7,14,15,16,17,18,19]. The implementation of appropriate interventions for specific patient populations has led to improved operative mortality and postoperative complication rates. A controversy remains surrounding which determinants to prioritise in preoperative assessment and decision-making to improve efficacy. Improved assessment of these factors may eliminate ambiguity regarding the risks of the procedural approach taken and predict operative mortality. The consideration of the impact of various surgical strategies on outcomes requires more reliable data. Similarly, further analysis is needed on the impact of the volume–outcome ratio of the surgeon or centre on mortality, which is poorly understood at present [1,2,7,20,21,22]. In this context, a greater comprehension of the factors influencing patient outcomes during surgical interventions can provide significant assistance to the decision-making process. This understanding can assist in guiding the selection of the most appropriate surgical option and ultimately improve the outcomes of those who reach specialist referral centres for the treatment of TAAAD. Additionally, accurate risk stratification can offer improved clinical assistance to patients and enable surgeons who treat patients during hospitalisation to be benchmarked. As there is limited information available in the TAAAD literature on the effects of serum lactate concentration, the current study seeks to examine this predictor in relation to the most frequently evaluated outcome predictors in individuals undergoing surgery for TAAAD. Specifically, we assessed whether preoperative arterial lactate levels elevate the risk of operative mortality in patients with acute type A aortic dissection (TAAAD), considering clinical and perioperative variables and surgical approach type.

## 2. Methods

This research is a component of a study approved by the Institutional Review Board (IRB ID 202201173) and is recognised by the NCT 05927090. Patient agreement has been revoked following research guidance. This study complies with the Declaration of Helsinki.

### 2.1. Data Extraction and Cleaning

Comprehensive analysis of prospectively collected data from three cardiovascular centres (Centre Cardiologique du Nord, Henri Mondor University Hospital, and the University of Genoa) was conducted. The Central Cardiac Database, which combines three independent databases for cardiovascular outcomes research, was retrospectively analysed in the DISC Department at the University of Genoa. The registry prospectively gathers demographic information, along with pre- and postoperative clinical data, encompassing mortality, for all TAAAD procedures carried out in three adult cardiac surgery centres in France and Italy. Previous reports have detailed the flow of data from input by surgeons to analysis, including different surgical approaches [7,23,24,25]. To summarise, the data were inputted locally and checked at the unit level by database managers before being uploaded to a single database for analysis. Reports were then generated for primary variables such as EuroSCORE risk factors, patient identifiers, and outcome data. The data were transferred to an academic health informatics department for data cleansing. A second statistician re-analysed the entire data collection process. Duplicate recordings were removed, transcriptional discrepancies recoded, and clinical and temporal conflicts resolved. The missing data were resolved during the data transfer validation phases from the individual centres.

### 2.2. Study Endpoints 

The primary endpoint was defined as operational mortality, which encompasses mortality within 30 days and during hospitalisation. Secondary endpoints include elevated arterial lactates upon hospitalisation, stroke, mesenteric ischemia, paraplegia, sepsis, dialysis, atrial fibrillation, reoperation for bleeding, sternal wound infection—mediastinitis, and VA ECMO. The operational strategy can be found in the supplementary material [1,7].

### 2.3. Statistical Analysis 

Categorical data were displayed as frequencies and percentages then compared utilising the χ^2^ test or Fisher’s exact test where suitable. Continuous variables were expressed as mean and standard deviation (SD) or median and interquartile range (IQR) if presenting normal or non-normal distribution. These variables were compared using a 2-tailed *t*-test or Mann–Whitney test. Imputation was performed if missing values were below 20%. We utilised predictive mean matching for the imputation of numerical features and logistic regression for binary variables. LASSO regression was applied to ascertain factors linked to early postoperative mortality, with an aim to minimise the possible collinearity of over-fitting variables and variables assessed from the same patient. The R package “glmnet” was employed for carrying out the LASSO regression. This is a logistic regression model that penalises the absolute size of the coefficients of a regression model using the value of λ. As the penalties increase, the estimates of weaker factors tend to shrink to zero, leaving only the strongest predictors in the model. The covariates with the most significant predictive power were chosen based on the λ (λ = Lambda.1se). Subsequently, variables identified through LASSO regression analysis were included in logistic regression models utilising stepwise variable selection based on the Akaike information criterion (AIC), and those that remained consistently statistically significant were used to formulate the risk model. The variance inflation factor (VIF) was used to check for collinearity among the independent variables. Discrimination was evaluated through the use of the receiver operating characteristic (ROC) curve and the area under the curve (AUC). Calibration was determined by comparing predicted mortality rates from the final model to actual observed rates. The R package “cutpointr” was utilised to calculate the “optimal” cutpoint (cutpoint estimation method maximising the metric function after spline smoothing and using 1000 bootstraps) of arterial lactate in forecasting initial mortality. Outcomes were categorised according to the cutpoint and adjusted for age, gender, renal function, hypertension, diabetes, peripheral arteriopathy, pulmonary disease, preoperative stroke, prior cardiac surgery, recent myocardial infarction, left ventricular ejection fraction, and family history of aortic diseases. Odds ratio, risk ratio, and risk difference were then calculated. To determine the risk factors associated with elevated arterial lactates, we conducted a thorough analysis to identify the most appropriate independent variables for predicting increased arterial lactates in multiple linear regression. The significance level was set for an alpha value of 0.05 [26,27].

The entire statistical analysis was carried out using R Statistical Software (version 4.3.1; R Foundation for Statistical Computing in Vienna, Austria).

## 3. Results

Of the 633 consecutive patients operated on for type A acute aortic dissection from January 2005 to December 2022, 449 patients with preoperative arterial lactate data were chosen for the present study’s patient population. The selection criteria included adults (aged >18 years) who underwent TAAAD or intramural haematoma surgery. The inclusion criteria stipulated the involvement of the lesion in the ascending aorta, occurrence of symptoms within seven days of surgery, and referral of patients for primary surgical repair of TAAAD in accordance with recommendations. Furthermore, other significant cardiac surgical procedures required during TAAAD and retrograde extension of TAAAD were taken into account. Patients under 18 years old, those with previous TAAAD procedures, delayed presentation (acute aortic syndrome >7 days prior), traumatic aortic dissections, and endocarditis were all excluded. Overall, 304 patients (67.6%) were male, and the mean age was 64 years. See Table 1 for complete demographic and clinical characteristics of the patients. 

All patients underwent aortic hemiarch replacement with an open distal anastomosis at a minimum. Primary entry tears occurred in 283 cases (63.0%) in the ascending aorta, 115 (25.6%) in the aortic root, and 59 (13.1%) in the aortic arch. Of the patients, 102 (22.7%) underwent total or partial arch repair. Preoperative blood lactate levels were 1.40 mmol/L [IQR 1.00, 2.80], and 131 (29.2%) had malperfusion syndrome before surgery. The patients were categorised into five groups by increasing haemodynamic severity. For those needing “urgent” intervention, the procedure was conducted within 24 h of admission during their initial hospital stay. Patients in this group had few symptoms with stable haemodynamics and no signs of malperfusion. Patients in the “emergency 1” group had symptoms, however, were stable from a haemodynamic perspective. They showed no indications of malperfusion or rupture, and surgery was advised within hours of being admitted. The “Emergency 2” grouping comprised patients who necessitated prompt intervention immediately after hospitalisation, or those who quickly worsened clinically. These patients exhibited haemodynamic instability that was not responsive to the administration of inotropes and/or malperfusion. Cardiopulmonary resuscitation is required for such categories of patients. “Salvage 1” and “Salvage 2” categories encompassed patients who required cardiopulmonary resuscitation with external chest compressions and/or open cardiac massage and were hospitalised as a result. These patients were in critical condition and were induced and initiated on cardiopulmonary bypass. “Salvage 1” patients underwent timely surgical procedures with cardiopulmonary bypass initiation due to worsening clinical conditions after anesthesia induction. They may have suffered from a cardiac tamponade, acute heart failure, or sudden rupture of the aorta. In the case of “Salvage 2” patients, cardiopulmonary resuscitation with external chest compressions was necessary during transport to the operating room or prior to anesthesia induction. Swift initiation of cardiopulmonary bypass was necessary, often preceded by cardiac massage after median sternotomy. The surgical procedure was completed without prior knowledge of genuine aortic rupture or severe organ damage.

### 3.1. Predictors of Early Postoperative Mortality

**Figure 1:**
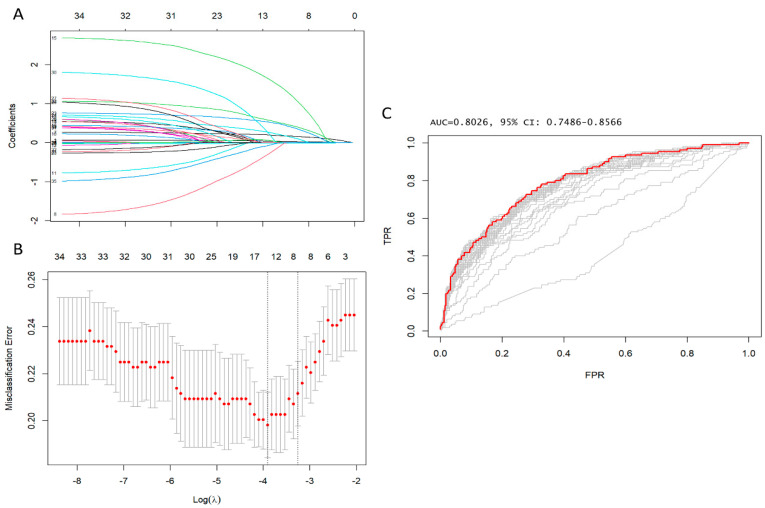
(**A**) Change of estimated coefficients with respect to the change of the penalty parameter. (**B**) cross-validation results with some proper range of log(λ) and MSE (mean squared error). The lambda value that minimises the test MSE turns out to be 0.02001626. (**C**) Cross-validated areas under the receiver operating characteristic curve (AUC). Model with best λ is red.

The operative mortality rate was 24.5% (110 out of 449 patients). No noteworthy difference in mortality rates was observed when comparing surgery with or without partial or total arch repair [83 out of 347 patients (23.9%) vs. 27 out of 102 patients (26.5%), *p* = 0.69] or aortic root replacement [26 out of 109 patients (23.9%) vs. 84 out of 340 patients (24.7%), *p* = 0.96]. In contrast, a significant difference was noted in cases where patients suffered from malperfusion [48 out of 131 patients (36.6%) vs. 62 out of 318 patients (19.5%), *p* < 0.001]. The elevation of lactate levels is a noteworthy predictor of premature death, irrespective of other factors (OR 1.3781, 95% CI 1.1756–1.6156, *p* = 0.0001). Please see Table 2 for additional information. In patients with malperfusion, there was a statistically remarkable increase in preoperative arterial lactates [2.00 mmol/L (IQR 1.20, 3.20 mmol/L) compared to 1.20 mmol/L (IQR 0.90, 2.20 mmol/L), *p* < 0.001]. No significant difference in early postoperative mortality was found when comparing patients who underwent surgery in the 1st half versus the 2nd half of the study period [26.0% (57 out of 219 patients) between 2005 and 2013 versus 23.0% (53 out of 230 patients) between 2015 and 2022, χ^2^ statistic = 0.54; *p* = 0.46]. A total of 36 variables measured at baseline (i.e., hospital admission) (Table 1) were included in the LASSO regression analysis. After LASSO regression selection (Figure 1, Supplementary Tables S1 and S2), the following 14 variables remained significant predictors of early postoperative mortality, including age, eGFR, arterial lactate, family history of aortic dissection or aneurysm, poor mobility, left ventricular ejection fraction, cardiac tamponade, preoperative intubation, malperfusion, cardiopulmonary bypass time, bicuspid aortic valve, antegrade cerebral perfusion, retrograde cerebral perfusion, and urgency of the procedure—Salvage grade 1 (procedure carried out in patients requiring cardiopulmonary resuscitation with external chest compressions and/or open-chest cardiac massage between the induction of anaesthesia and the initiation of cardiopulmonary bypass).

These 14 variables were included in the logistic regression model, 8 variables were retained, and 7 variables were identified as noteworthy independent predictors of operative mortality. These variables included age, eGFR, arterial lactate, poor mobility, cardiac tamponade, preoperative intubation, and cardiopulmonary bypass time (Table 2). 

**Figure 2:**
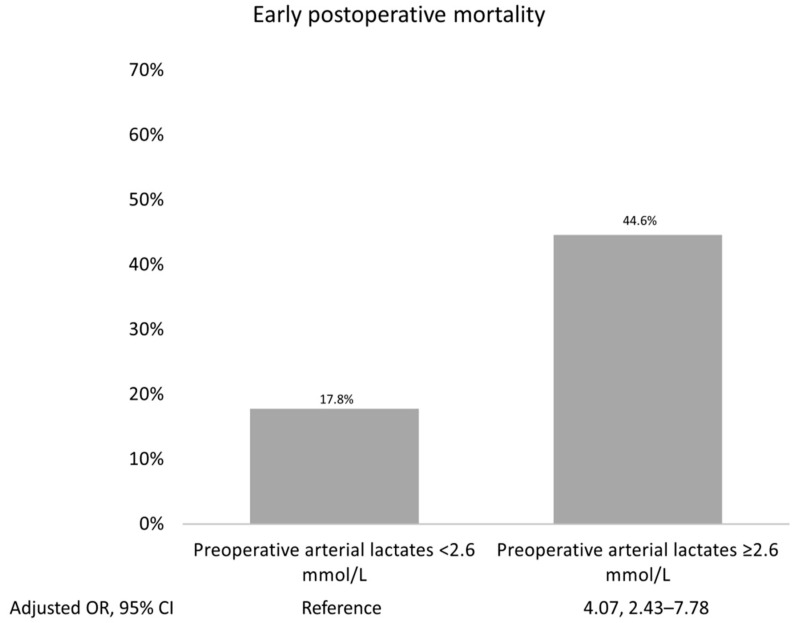
Early postoperative mortality stratified for arterial lactates exceeded the prognostic cut-offs of 2.6 mmol/L. Odds ratios adjusted for age, gender, renal function, hypertension, diabetes, peripheral arteriopathy, pulmonary disease, preoperative stroke, prior cardiac surgery, recent myocardial infarction, left ventricular ejection fraction, and family history of aortic diseases have been reported.

Furthermore, the area under the ROC was 0.84 (95% CI: 0.79–0.88), Hosmer–Lemeshow’s test *p* value was 0.48, and, VIF was <1.5, indicating no collinearity amongst all the independent variables (Supplementary Table S3). The most accurate prognostic cut-off level of arterial lactate for forecasting early postoperative mortality was determined to be ≥2.6 mmol/L, with an ROC curve value of 0.67. A sensitivity of 45%, specificity of 38%, and an Adjusted OR of 4.07 (95% CI 2.43, 7.78; *p* < 0.001) were noted. The results are illustrated in the prognostic ROC curves (Supplementary Figure S1 and Supplementary best cut-off statistics, Table S3). Figure 2 shows the highest early postoperative mortality rate that occurred when arterial lactates exceeded their prognostic cut-offs.

### 3.2. Early Postoperative Outcomes 

Table 3 presents a breakdown of early adverse outcomes following surgery based on the preoperative arterial lactate cut-off (≥2.6 mmol/L). In addition to a higher early postoperative mortality, elevated lactate levels result in increased crude rates of postoperative mesenteric ischemia (11% vs. 3.9%, *p* = 0.03), sepsis (33.9% vs. 21.4%, *p* = 0.01), and VA ECMO (12.5% vs. 2.7%, *p* < 0.001). After adjusting for baseline characteristics, it was confirmed that preoperative arterial lactates ≥ 2.6 mmol/L were associated with postoperative dialysis and the need for VA ECMO implantation. The adjusted risk differences were 9.3% and 7%, respectively.

### 3.3. Predictors of Increased Preoperative Arterial Lactates 

**Figure 3:**
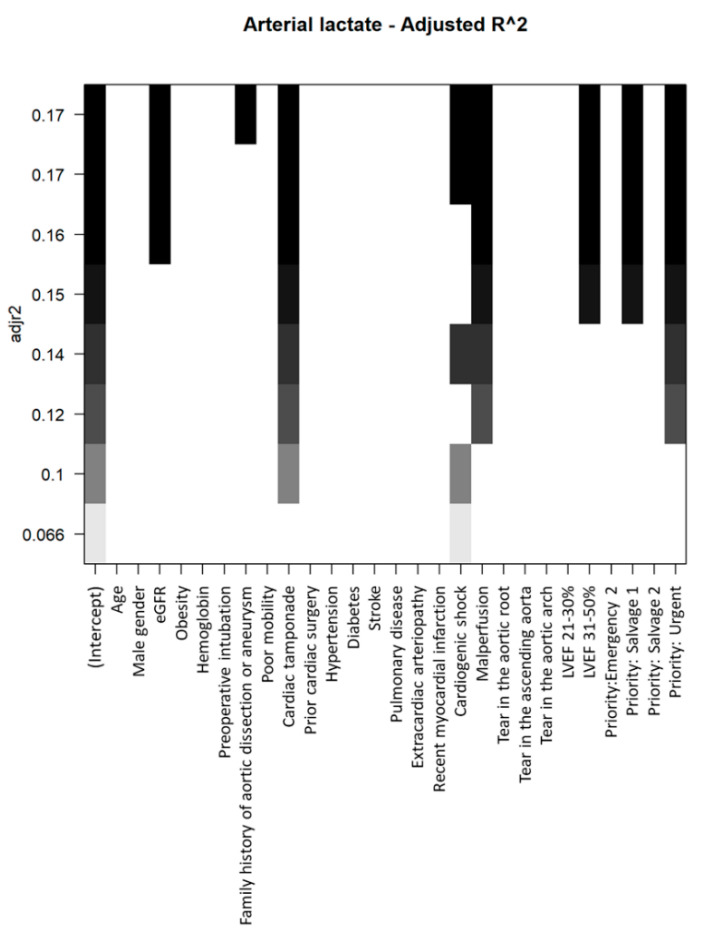
Selection process of demographic and clinical variables to be included in the multivariable model for predicting increased arterial lactate levels.

The process of selecting baseline variables to be incorporated into multivariable models for elevating preoperative arterial lactates is illustrated in Figure 3. 

Within the multivariable model, eGFR, cardiac tamponade, cardiogenic shock, LVEF 31–50%, and the urgency of the procedure (Urgent and Salvage grade 1) were identified as independent factors associated with increased preoperative arterial lactates (refer to Table 4).

## 4. Discussion

Significant progress has been made in improving diagnosis using imaging modalities and the medical treatment of TAAAD since its inception. The introduction of drug therapy, including the application of β-blockers, as well as the emergence of sophisticated and diverse cardiovascular, vascular, and hybrid surgical alternatives, have propelled the care process for patients with TAAAD forward [3,28,29,30,31,32,33]. In this detailed analysis, we assessed the survival rates post-surgical repair for acute type A aortic dissection in patients with malperfusion and raised serum lactate levels. The study is founded upon a 17-year span (2005–2022) of amassed data from a multi-centred registry comprising three esteemed centres.

Our findings demonstrate that for patients with TAAAD, the unadjusted operative mortality rate was 24.5%, showing no significant variation between those who received partial or total arch repair or aortic root replacement. Our 17 year analysis reveals that the surgical approach to TAAAD continues to result in high mortality, as indicated by multiple multi-centred registries; however, the mortality rate is gradually decreasing [3,8,14,15,16,17,18]. Furthermore, we have identified seven distinct risk factors linked to early mortality: age, arterial lactate, eGFR, cardiac tamponade, preoperative intubation, cardiopulmonary bypass time, and poor mobility. These factors have been incorporated into a bedside risk scoring tool primarily based on arterial lactate serum levels. 

A surgical strategy for TAAAD has been associated with reduced rates of operative mortality. This ranges from 16–18% for patients who received root-sparing AAR using Dacron graft interposition with or without hemiarch repair [3,8,14,15] to 26.9% for those who underwent TARP [16]. However, various centres report varying percentages for lower operative mortality. The initial 48 h following the emergence of clinical symptoms associated with aortic dissection mark a critical period for those suffering from TAAAD due to their increased susceptibility to malperfusion. Such individuals may exhibit high levels of serum lactate linked to malperfusion prior to their admission to the hospital or even before the diagnosis of aortic dissection is made. 

The findings detailed in this report align with a notable rise in patient mortality rates, particularly in those with elevated preoperative serum lactate levels. To ensure the accuracy of the study, we established five distinct categories of heightened haemodynamic severity [28], factoring in the time of the initial aortic dissection and subsequent hospitalisation. By doing so, we were able to group patients according to arterial lactate value increments and assess mortality rates. For these patients, all of whom were considered candidates for surgery, it was observed that in the presence of high arterial lactate levels, the difference in risk mortality increased significantly by 23.4%. Furthermore, the risk mortality rate remained disappointingly high with an arterial lactate cut-off of ≥2.6 mmol/L (13.8% vs. 32.9%; *p* < 0.00).

Our findings indicate that the mortality rate of the patient within the first 48 h after the surgery, as well as the complications arising during ICU surveillance within the initial days following surgery, were consistent with higher preoperative arterial lactate levels. Alongside the risk of operative mortality, elevated arterial lactate levels are correlated with a substantial post-operative increase in the likelihood of VA ECMO (7%; *p* < 0.001) and the need for dialysis (9.3%; *p* = 0.003). The results indicate that age, cardiac tamponade (*p* < 0.0116), and preoperative intubation due to haemodynamic instability and hypotension (*p* = 0.005) were significant predictors of mortality following surgery. While the mortality rate for surgical procedures has significantly decreased in recent years, the estimate for in-hospital mortality in patients who receive optimal medical treatment (OMT) or suffer from malperfusion-related clinical complications remains constantly high. Patients undergoing OMT have a mortality rate greater than 50% [34], whereas patients with arterial lactate cut-off ≥ 2.6 mmol/L have a mortality rate of 32.9%, as observed in this study (*p* < 0.001). For individuals diagnosed with TAAAD, it is important to consider both the in-hospital mortality rate and the mortality rate in the wider population. It is worth noting that our study’s findings only account for patients who died whilst in the tertiary care centre and do not include those who died before arriving at the centre, including those transferred from other hospitals. Our findings correspond with those reported in the IRAD registry [3] and the UK National Adult Cardiac Surgical Audit [8]. The mortality rate for TAAAD in the wider population is substantially higher [6].

### 4.1. Malperfusion Management Subgroup

There exists a subgroup of patients deemed to carry an unmanageable degree of risk for operative mortality and post-operative complications that are serious and irreversible. This assessment is established purely on the grounds of evidence provided by various stakeholders, namely physicians/surgeons, patients, and family members. This diverse cohort includes elderly individuals, those with significant coexisting conditions, and those who only satisfy emergent clinical criteria for TAAAD repair [1,20]. Our appraisal of the IRAD registry demonstrated an upward trend in surgical interventions, with 90% of patients currently undergoing TAAAD repair. Importantly, a segment of this group of individuals declined to undergo surgical intervention [3,29,30,31,32]. As in prior studies, we found a subgroup of patients who initially presented with indications of cerebrovascular, mesenteric, and peripheral ischemia tied to acute and severe clinical situations, where the urgency of the procedure was deemed necessary. Along with advanced age and previous cardiac surgery, malperfusion findings are well-established markers of escalated operative risk and determine the decision-making process that guides TAAAD repair [1,10,33,34].

There is a strong link between malperfusion and a potential rise in arterial lactate levels. It is important to note that the definition of malperfusion can differ depending on the assessed registers. In our analysis, we grouped patients who experienced mesenteric malperfusion, renal malperfusion, cardiogenic shock (including cardiac tamponade and low cardiac output), any pulse deficit/limb ischemia, and cerebral perfusion deficit (recent cerebrovascular accident) as having organ malperfusion. We reported a rate that was comparable to that described in the GERAADA database [8] in the UK and higher than that reported in the NORCAAD registry [11]. In our study, although an aortic tear was found in all patients, only 30.5% of cases presented subclinical or symptomatic malperfusion due to the static or dynamic behavior of the aortic tear. This led to multi-organ ischemia with increased arterial lactate and systemic metabolic damage [1,3,35,36,37].

Malperfusion persists when the true lumen of a vessel originating from the aortic conduit is obstructed by the expansion of a haematoma or thrombosis in the false lumen or by the action of the dissecting membrane itself. In such circumstances, timely and effective recanalisation is essential. It is possible that persistent poor dynamic perfusion could result in a vicious cycle that ultimately leads to a high lactate cutoff. In these situations, the membrane hovers in front of the aortic branch or protrudes into a branch, causing obstructive dynamics and consequent pressure fluctuations in the genuine and fake lumens during the cardiac cycle. We noticed a rise in raw rates of mesenteric postoperative ischemia (11% vs. 3.9%, *p* = 0.03) because of the continual disruption of regional circulation in patients whose arterial lactate exceeded 2.6 mmol/L; this may be linked to continual branch thrombosis caused by low flow and embolism resulting in malperfusion. In our analysis, we noted that arterial lactate levels were elevated in cases of renal malperfusion (*p* = 0.009) along with cardiac tamponade (*p* < 0.0001), cardiogenic shock (*p* = 0.05), LVEF (*p* = 0.014), and priority urgency procedures (salvage 1 *p* = 0.05; urgent *p* = 0.0001). Therefore, after determining preoperative risks, a meticulous clinical examination, as well as surgical centre proficiency, becomes particularly crucial in high-risk cases [8,24,38].

Although strong evidence has established that TAAAD repair may be a viable surgical option for high-risk groups such as octogenarians, offering TAAAD repair to these patients requires a balance between increased operative risks and high mortality rates. Rapid and informed decision-making should be made in the presence of malperfusion associated with higher lactate cut-offs while considering patients and their families [1,30,36]. On the contrary, the cut-off for serum lactate below 2.6 mmol/L appears to substantiate the findings of past studies, which recorded a lower mortality rate that did not surpass 7% [10,11,12,13], approximately half that of the International Registry of Acute Aortic Dissection, German Registry for Acute Aortic Dissection Type A, Nordic Consortium for Acute Type A Aortic Dissection, and UK National Adult Cardiac Surgical Audit [3,8,14,15]. It is possible that these patients were not referred for TAAAD repair despite worsening organ malperfusion, which may have eliminated the molecular impacts of heightened arterial lactate concentration on OM and postoperative outcomes.

### 4.2. Surgical Repair in Patients with Higher Lactate

The optimal treatment plan for TAAAD in patients with heightened arterial lactate must balance the need for low operative mortality during the index procedure with minimal risk of future reoperation. However, if the ideal surgical option requires increased procedural complexity, it must be carefully considered, as this may lead to prolonged periods of cardiac and organ ischemia during surgery. Our research suggests that the duration of cardiopulmonary bypass is a contributing factor to the heightened occurrence of OM (*p* = 0.0011), evidenced by the significant coefficient in the LASSO logistic regression model for OM (So = 0.0023). Therefore, when monitoring TAAAD repair, it is advisable to keep the arterial lactate level below the cut-off, as this is identified as an additional risk factor that can lead to multi-organ failure. This notion is supported by aggregated multicentre data. For instance, data published by the Society of Thoracic Surgeons for the period between 2014 and 2016 showed an overall mortality (OM) rate of 26.9% among recipients of total arch replacement, in comparison to an OM rate of 16.3% in patients who underwent hemiarch repair [39]. In a secondary report utilising pooled meta-analytical data, a comparison was made between proximal conservative repair and extended arch repair. The analysis revealed that in the former approach, the risk of early mortality was reduced (relative risk, 0.69) in comparison to the likelihood of requiring a distal reoperation (relative risk, 3.14).

To the best of our knowledge, this is the most extensive evaluation of factors that affect outcomes following surgical repair for TAAAD and establish a threshold for the arterial lactate. Although arterial lactate is an indicator of tissue ischemia, there is a lack of data regarding the determination of the arterial lactate threshold in the scientific literature on TAAAD, and no assessment is reported on the relationship between arterial lactate fluctuations and organ malperfusion, which requires significant attention.

This issue contributes to the uncertainty surrounding the inconsistent outcomes regarding operative mortality as seen in numerous single-centre studies. It is possible that this result is caused by the worsening clinical status of patients due to malperfusion and multi-organ systemic metabolic damage. In one study, it was suggested that there was an increase in operative mortality for patients undergoing TAAAD repair, with the ascending aorta having a mortality rate of 9.8%, the hemiarch having a mortality rate of 21.6%, and TARP having a mortality rate of 28.6%. In contrast, another study reported slightly lower rates of OM (13.4% vs. 9.7%) in recipients of hemiarch repair compared to those who received total arch replacement. However, the authors noted a significantly higher incidence of cerebral malperfusion and permanent neurological deficit in the total arch replacement group (22.7% vs. 6.3%).

### 4.3. Tailored Surgery 

In our study, the majority of patients underwent limited replacement of the ascending aorta using an interposition graft, with or without hemiarch repair. This surgical approach was succeeded by aortic root replacement, whereas only a minor proportion of cases had TARP performed. The mortality rate showed no significant variation post-surgery, whether it included partial or total arch repair (*p* = 0.69) or aortic root replacement (*p* = 0.96). However, it was observed that the operative mortality was likely influenced by independent predictors such as cardiopulmonary bypass time and antegrade cerebral perfusion.

These findings support the theory that a total aortic arch replacement, which entails an extended period of ischemia, should only be carried out after evaluating the patient’s preoperative state, monitoring arterial lactate levels, and reviewing surgical outcomes. Likewise, supporters of conservative surgery recommend complete removal of the intimal lesion along with the implant of a Dacron prosthesis to replace the ascending aorta with or without hemiarch. These options are still considered to be the most commonly used surgical techniques for TAAAD, as confirmed by sources [3,8,10,12,13,14,15,16].

In this study, the surgical repair extent (ascending aorta, hemiarch, total arch replacement) mainly addressed critical clinical conditions upon admission, such as malperfusion and arterial lactate concentration, and the characteristics of the aortic lesion, to minimise the risks of operative mortality and postoperative complications. The primary purpose remains the surgical repair of the entry tear of the dissection. Therefore, for patients with an entry tear that affected the lesser curvature of the aortic arch, we opted for the conservative surgical approach with the use of a Dacron prosthesis and hemiarch repair. Additional surgical options may have to be considered in cases of entrance lesions near the supra-aortic branches, in line with the degree of aortic arch involvement and preoperative patient conditions. These options may include more extensive arch surgery, as appropriate.

In recent years, there has been significant progress in providing aortic pathology services as a subspecialty. Advancements in surgical techniques, cerebral protection, and the introduction of new technologies have encouraged extensive surgery for TAAAD repair. This involves replacing the aortic arch with potential extensions into the proximal descending aorta, along with hybrid procedures. The advantages of expanding the initial TAAAD operation to encompass treating extra dissected aortic portions become apparent through a higher level of risk reduction in the advancement of aortic dilation and rupture of the resected aortic aorta by means of second-phase endovascular treatment alternatives. However, although this anticipated medium and long-term advantage has been noticed for extensive repairs, it is imperative to carefully consider the risk of potentially higher mortality and morbidity resulting from the greater complexity of the initial procedure [40,41,42]. Moreover, there is further evidence of percentage disparities in the outcomes for TAAAD repair in patients undergoing TARP or TARP plus FET [43,44,45,46]. Proponents of more extensive surgical options compared recipients of conservative repair with those who underwent the extended TARP procedure. The incidence of OM (24.1% vs. 22.6%) and PND (9.1% vs. 7.5%) was found to be similar in both groups [45]. It was also suggested that there were no differences in operative mortality (5.4% vs. 5.7%) or PND (1.4% vs. 2.3%) between conservative isolated hemiarch repair and FET [46]. However, there were concerns about a higher incidence of OM. In actuality, few centres support a more conventional surgical approach or the adoption of aggressive methods such as TAR, antegrade stent graft, or FET, which have consistently reported mortality rates below 10% [10,11,12,13,41]. However, TAR combined with FET constitutes a feasible strategy for mending the aortic arch and deserves consideration when analysing the outcomes emphasised by Sun and colleagues [13], who advocate strongly for this more comprehensive surgical approach in repairing TAAAD. They presented impressive findings from a cohort of 148 patients diagnosed with ruptured arch, arch aneurysm, or Marfan syndrome complicated by TAAAD who received TARP alongside FET. There was no significant difference in in-hospital mortality (4.7% vs. 6.1%; *p* = 0.74), stroke (2.7% vs. 1.5%; *p* = 1), and spinal cord injury (1.4% vs. 0; *p* = 1) when comparing TARP plus FET and hemiarch substitution. Additionally, the FET group exhibited an improvement in false lumen thrombosis and a decrease in the need for reoperations (1 vs. 4 patients; *p* = 0.03).

Our analysis found that patients who underwent total replacement of the aortic arch faced a higher probability of stroke when their arterial lactate cut-off was ≥2.6 mmol/L (9.2% vs. 14.3%; risk ratio 1.465) and a greater risk of operative mortality at Lasso logistic regression in patients who needed antegrade cerebral perfusion (s0 = 0.0498). Total aortic arch replacement has been proven to be a secure surgical choice in elective surgery. However, there is still debate around its impact on surgical mortality and morbidity and its long-term benefits in TAAAD repair [42]. Our results reinforce the hypothesis that TARP is a surgical option that requires assessment depending on the patient’s preoperative condition, given good surgical outcomes and a lower risk of postoperative complications. However, there is still debate around its impact on surgical mortality and morbidity, and its long-term benefits in TAAAD repair [42].

The procedure involving complete replacement of the aortic root was carried out in 24.3% of patients in our case series, without affecting the recorded mortality rate. Aortic root surgery in the context of TAAAD is technically complex and increases the risk of surgery. Patients in need of this more complicated procedure experience much longer bypass and cross-clamp times in comparison to those who undergo ascending aortic replacement with preservation of the root. In particular, the delicacy of the dissected aortic tissue may complicate the reimplantation procedure of the dissected coronary buttons. Furthermore, there is potential for postoperative bleeding and ischemia from coronary button malposition, which can be fatal. In cases of extensive root destruction, concomitant root aneurysm, bicuspid aortopathy, or a history of connective tissue disease, we have performed root replacement with equivalent operative outcomes, as reported in other studies [3,7,14,47,48].

### 4.4. Processes of Care and Aortic Centres 

The group of patients with malperfusion and elevated arterial lactate concentrations requiring critical hospitalisation for surgery underlines the significance of expediting TAAAD identification and patient transfer to surgery [37]. The study IRAD demonstrates ongoing delays in TAAAD recognition and treatment, with an average diagnosis time of 2.5 h and surgery time of 3.5 h following presentation. A timeframe of 6 h is generally needed from arrival in the emergency department to surgery. Additionally, some patients unfortunately pass away before undergoing surgery, with a median estimated time (IQR) from admission to death of 8.9 h [4,49]. At our centre, there is frequently no need for interhospital transfer, which limits therapeutic delays due to the implementation of a regional transfer process in Ile de France. This process entails the patient being transported directly to the operating theatre upon arrival.

The regional care model, which highlights diagnosis and treatment protocols, requires evaluation to demonstrate reduced diagnosis and treatment times. Chikwe and colleagues [50] claim that improved aortic dissection survival can be attributed to both surgeon and centre volume. Therefore, prompt referral to aortic centres with cardiovascular and vascular specialists, and anaesthetists adept in intricate aortic surgery, cerebral safeguarding, and methods to tackle malperfusion, are vital for these patients. Several studies have reported that transfer to high-volume specialised aortic centres reduces malperfusion complications and mortality [21,51,52,53,54]. Although hospital care system factors are critical for prompt diagnosis and treatment, it is equally important to consider the time from patient symptom onset to presentation, with a median time of 1.5 h [0.8–3.3] (IQR). Therefore, it is vital to educate patients so that those who are at risk can identify the symptoms of TAAAD, report quickly to the emergency department, and request targeted aortic testing where necessary [49,54].

## 5. Limitations

Although this study is the most comprehensive and up-to-date assessment of operative mortality and postoperative complication rates caused by increased arterial lactate concentrations in TAAAD patients, it has certain limitations that must be considered. The retrospective nature of the analysis and the incomplete or missing event information should be taken into account. Furthermore, the recorded survival rates based on arterial lactate levels are only applicable after TAAAD malperfusion recognition and referral for surgery. As Howard et al. [6] demonstrate, a significant proportion of patients with TAAAD do not survive their hospital stay. While malperfusion is identified as an independent risk factor in most registries, the exact arterial lactate thresholds are not reported. Therefore, the early mortality rates for patients undergoing TAAAD repair who are referred to tertiary centres are not known, and this is linked with multi-organ metabolic damage induced by malperfusion and sustained by the high concentration of arterial lactates. Finally, given the retrospective nature of the analysis, it is difficult to determine whether high arterial lactate values may be associated with the observed postoperative complications and mortality depending on the type of surgical option used, which dictated a conservative approach or an extensive approach.

## 6. Conclusions

Patients with a recognised TAAAD in contemporary times exhibit a mortality rate of 0.12% per hour during the first 48 h [30]. Despite significant advancements in surgery, medical care, and imaging over the past 60 years, leading to a substantial decline in mortality rates, the severe clinical status of patients sustained by systemic malperfusion results in the remaining high mortality rate. The arterial lactate concentration of patients referred for TAAAD repair is an independent factor influencing operative mortality and postoperative complications. Considering an arterial lactate threshold of 2.6 mmol/L, mortality increases by 23.4% for higher levels (risk ratio 2.264; 1.660–3.082). In addition to surgical mortality, the risk of VA ECMO and the need for dialysis is significant in patients who have an upper arterial lactate threshold of ≥2.6 mmol/L within 48 h after surgery. As arterial lactate, cardiopulmonary bypass, and anterograde cerebral perfusion are all independent factors that increase operative mortality, a tailored approach should be considered for patients with a higher arterial lactate cut-off of ≥2.6 mmol/L. This approach should dictate a more conservative option. These findings serve as a reminder that malperfusion in TAAAD requires more robust efforts to improve recognition, early initiation of transfer, and the surgical treatment protocol.

## Figures and Tables

**Table 1:** Preoperative clinical and operative characteristics.

Variables	N = 449
Age (median [IQR])	65.00 [56.00, 75.00]
Male	304 (67.7)
Weight, kg (median [IQR])	77.00 [69.00, 85.00]
Height, cm (median [IQR])	172.00 [165.00, 178.00]
Obesity (%)	67 (14.9)
eGFR, mL/min/1.73 m^2^ (median [IQR])	72.00 [56.00, 87.75]
Haemoglobin, g/L (median [IQR])	122.00 [108.00, 136.00]
Arterial lactate, mmol/L (median [IQR])	1.40 [1.00, 2.80]
Family history of aortic dissection or aneurysm (%)	31 (6.9)
Prior cardiac surgery (%)	17 (3.8)
Hypertension (%)	350 (78.0)
Diabetes (%)	25 (5.6)
Stroke (%)	6 (1.3)
Pulmonary disease (%)	24 (5.3)
Extracardiac arteriopathy (%)	21 (4.7)
Poor mobility (%)	11 (2.4)
Recent myocardial infarction (%)	17 (3.8)
Left ventricular ejection fraction (%)	
>50%	278 (61.9)
21–30%	8 (1.8)
31–50%	163 (36.3)
Systolic pulmonary artery pressure (%)	
<30 mmHg	365 (81.3)
<31 mmHg	2 (0.4)
>55 mmHg	8 (1.8)
30–55 mmHg	74 (16.5)
Bicuspid aortic valve (%)	9 (2.0)
Cardiogenic shock requiring inotropes (%)	61 (13.6)
Cardiac tamponade (%)	65 (14.5)
Preoperative intubation (%)	171 (38.1)
Any malperfusion excluding myocardial malperfusion (%)	131 (29.2)
Tear in the aortic root (%)	115 (25.6)
Tear in the ascending aorta (%)	275 (63.0)
Tear in the aortic arch (%)	59 (13.1)
CABG (%)	40 (8.9)
Ascending aorta replacement	238 (53)
Aortic root replacement (%)	109 (24.3)
Total or partial aortic arch repair (%)	102 (22.7)
Antegrade cerebral perfusion (%)	107 (23.8)
Retrograde cerebral perfusion (%)	203 (45.2)
Myocardial ischemic time, min (median [IQR])	118.00 [86.00, 166.00]
Cardiopulmonary bypass time, min (median [IQR])	205.00 [155.00, 273.00]
Hypothermic circulatory arrest duration, min (median [IQR])	39.00 [26.00, 54.75]
Urgency of the procedure *	
Emergency 1	100 (22.3)
Emergency 2	125 (27.8)
Salvage 1	19 (4.2)
Salvage 2	3 (0.7)
Urgent	202 (45.0)

CABG, coronary artery bypass grafting; eGFR, estimated glomerular filtration rate; IQR, interquartile range. * Urgent: scheduled procedure performed in paucisymptomatic patients with stable haemodynamic conditions during the index hospitalisation since the next working day from admission; Emergency grade 1: procedure performed in patients with stable conditions and without malperfusion before the beginning of the next working day; Emergency grade 2: Procedure performed in patients with haemodynamic instability despite the use of inotropes and/or any malperfusion before the beginning of the next working day—no cardiopulmonary resuscitation with chest compression required; Salvage grade 1: procedure performed in patients requiring cardiopulmonary resuscitation with external chest compressions and/or open chest cardiac massage between induction of anesthesia and initiation of cardiopulmonary bypass; Salvage grade 2: procedure performed in patients requiring cardiopulmonary resuscitation with external chest compressions en route to the operating theatre or before induction of anesthesia.

**Table 2:** Multivariable logistic regression for early mortality based on LASSO regression.

Variables	ODDS RATIO	95% CI	*p* Value
Age	1.0578	1.0321–1.0842	<0.0001
eGFR	0.9784	0.99662–0.9908	0.0007
Arterial lactate	1.3781	1.1756–1.6156	0.0001
Family history of aortic dissection or aneurysm	0.2343	0.0534–1.0281	0.0545
Poor mobility	14.0323	2.6177–75.2198	0.0020
Cardiac tamponade	2.3044	1.2053–4.4056	0.0116
Preoperative intubation	2.3994	1.4174–4.0617	0.0011
Cardiopulmonary bypass time	1.0044	1.0017–1.0070	0.0011

CI, confidence interval; eGFR, estimated glomerular filtration rate; IQR, interquartile range. AUC: 0.8307 95% CI: 0.7861–0.8753; Hosmer and Lemeshow Goodness-of-Fit Test *p* = 0.47.

**Table 3:** Early postoperative outcomes stratified for arterial lactate ≥2.6 mmol/L.

Variables	Arterial Lactate <2.6 mmol/L (N = 337)	Arterial Lactate ≥2.6 mmol/L (N = 112)	*p* Value	Risk Difference (95% CI) *	Risk Ratio (95% CI) *	Odds Ratio (95% CI) *
Early mortality	60 (17.8%)	50 (44.6%)	<0.001	23.4% (13.8%, 32.9%)	2.264 (1.660, 3.082)	4.069 (2.430, 7.783)
Stroke	31 (9.2%)	16 (14.3%)	0.179	4.3% (−4.2%, 11.3%)	1.465 (0.620, 2.466)	1.557 (0.581, 3.051)
Paraplegia	10 (3.0%)	3 (2.7%)	1.000	0.3% (−2.3%, 5.6%)	1.115 (0.000, 3.593)	1.121 (0.000, 4.322)
Mesenteric ischemia	13 (3.9%)	11 (9.8%)	0.029	5.0% (−1.3%, 9.2%)	2.235 (0.747, 4.984)	2.547 (0.702, 6.886)
Sepsis	72 (21.4%)	38 (33.9%)	0.011	8.2% (−3.1%, 18.8%)	1.367 (0.872, 2.028)	1.560 (0.824, 2.903)
Dialysis	40 (11.9%)	27 (24.1%)	0.003	9.3% (2.0%, 16.7%)	1.743 (1.157, 2.576)	2.077 (1.228, 3.460)
Atrial fibrillation	81 (24.0%)	24 (21.4%)	0.663	−4.7% (−12.6%, 3.9%)	0.808 (0.507, 1.185)	0.752 (0.403, 1.260)
Reoperation for bleeding	38 (11.3%)	10 (8.9%)	0.603	−2.8% (−9.9%, 5.3%)	0.753 (0.244, 1.641)	0.713 (0.189, 1.960)
Deep sternal wound infection—mediastinitis	11 (3.3%)	8 (7.1%)	0.135	1.0% (−2.2%,5.2%)	1.250 (0.479, 2.925)	1.287 (0.453, 3.870)
VA ECMO	9 (2.7%)	14 (12.5%)	<0.001	7.0% (1.3%, 14.3%)	3.360 (1.343, 10.164)	3.949 (1.428, 17.172)

CI, confidence interval; VA ECMO, veno-arterial extracorporeal membrane oxygenation. * Outcomes were adjusted for age, gender, renal function, hypertension, diabetes, peripheral arteriopathy, pulmonary disease, preoperative stroke, prior cardiac surgery, recent myocardial infarction, left ventricular ejection fraction, and family history of aortic pathologies.

**Table 4:** Predictors of increased arterial lactate.

Independent Variables	Beta	t	*p* Value
eGFR	−0.0101	−2.611	0.009
Cardiac tamponade	1.0516	4.240	<0.0001
Cardiogenic shock	0.6409	1.968	0.05
LVEF 31–50%	0.4499	2.451	0.014
Priority: Salvage 1	1.1128	1.968	0.05
Priority: Urgent	0.8814	3.892	0.0001

R^2 = 0.18; R^2 Adjusted = 0.17; SEE = 1.776. Beta: standardised regression coefficients.

## Data Availability

Nappi, Salsano, and Fiore were granted complete access to all data in the study and acknowledge their responsibility for the data’s integrity and accuracy of the data analysis. Requests for the data supporting this article will be fulfilled upon reasonable inquiry to the corresponding author.

## Supplementary Materials

The following supporting information can be downloaded at: https://www.mdpi.com/article/10.3390/jcm12196177/s1, Table S1. Coefficients of the best model of LASSO logistic regression for early mortality; Table S2. Accuracy of LASSO REGRESSION; Table S3. Variance inflation factor calculation for each inde-pendent variable of multiple logistic regression; Figure S1. The top left plot shows predictor values per class, the top right shows the receiver operating characteristic curve (ROC curve), the bottom left showing the bootstrapped cutpoint variability, and the bottom right showing the distribution of the out-of-bag metric values (see best cut-off statistics in supplemental materials for details).
