# Supplementary material for: Lactate-Based Difference as a Determinant of Outcomes following Surgery for Type A Acute Aortic Dissection: A Multi-Centre Study
Source: J Clin Med. 2023 Sep 25;12(19):6177. doi: 10.3390/jcm12196177 (PMC10573384; doi:10.3390/jcm12196177)
Supplement: Supplementary file 1 [file jcm-12-06177-s001.zip › jcm-2622923-supplementary.pdf]

Supplementary Material

Surgical technique

Sternotomy was undertaken for all patients, with the lead surgeon of each institution directing the operative strategy for preferred cannulation site. Arterial cannulation was carried out either in the right common femoral artery, brachiocephalic trunk, axillary artery, or centrally via the aortic lumen. Surgical preference dictated the level of systemic cooling. Cardiopulmonary bypass was initiated simultaneously with systemic cooling. Potassium-rich antegrade cardioplegia solution was administered into the coronary ostium to achieve diastolic arrest. In cases where TAAAD induced aortic regurgitation or when patients necessitated more radical aortic root replacement procedures or extensive repairs involving the aortic arch, a coronary sinus perfusion cannula was introduced to deliver the cardioplegic solution retrogradely. The sinotubular junction served as a reference point for the removal of the ascending aorta. Typically, thrombi would occupy the false lumen of the aortic root and/or ascending aorta, and their removal allowed for a more accurate assessment of the extent of aortic damage. This was followed by thorough examination of the anatomical soundness of the aortic root and leaflets. 4-0 or 5-0 sutures, with Teflon pledgets, were employed to stitch the tunica intima of the commissures to the adventitia, reinforcing each commissure for resuspension. Such a method has been implemented in patients requiring both root sparing - ascending aorta replacement (AAR) and those undergoing root replacement - AAR in cases where the intimal detachment had extended into the sinuses of Valsalva, eventually leading to commissural collapse. Normally, during the reconstruction of the aorta, neo-media biological glue is applied, while the use of felt is based on surgical preference. A 4-0 or 5-0 polypropylene suture can seal the proximal suture line, and this anastomosis can also buttress the two intimal and adventitial walls to ensure solid continuity. Some surgeons apply felt as the neo-media or use horizontal felt-mattress sutures arranged circumferentially to achieve a continuous outer ring of felt reinforcement. Ascending root replacement was conducted through biological or mechanical composite valve grafts or via a valve-sparing root replacement procedure governed by the sinuses of Valsalva, as identified through computed tomography utilizing 4.5 cm as a cut-off. Alternatively, if intimal tears were observed in the sinus of Valsalva or in patients with connective tissue disease. Conversely, for patients with non-dilated aortic roots but poor-quality valve leaflets, the recommended surgery is to replace the aortic valve with a bioprosthetic or mechanical prosthesis and place a Dacron graft.

In patients necessitating total arch replacement procedure (TARP), deep hypothermic circulatory arrest (DHCA) was applied along with either continuous antegrade cerebral perfusion or retrograde cerebral perfusion with systemic cooling to a temperature of 19°C or 25°C, depending on surgical preference. The brain's symmetrical cooling and re-warming were continuously observed with near-infrared spectroscopy. 80.5% of patients who underwent TARP needed a protocol for antegrade brain protection. This was achieved using either endoluminal techniques or direct insertion of the cannula into the brachiocephalic trunk, left common carotid artery, or right axillary artery. A different flow rate was delivered ranging from 800 to 1,000 mL/min when the temperature was fixed at 28°C or 36°C and the systemic arterial pressure was maintained between 40- 60 mmHg. The remaining TARP (19.5%) utilized DHCA by retrograde cerebral perfusion via a superior vena cava cannula. The delivery of the solution was set at 200-350 mL/min at 18°C and a target central venous pressure of between 25-35mmHg.

TARPs comprised of partial or complete resections. Both 1- and 4-branch grafts were utilized for entirely removing the aortic tissue up to zone 2, which included the left common carotid artery (total hemiarch), as well as for less comprehensive resections (partial hemiarch) in patients who received reimplantation of only the brachiocephalic trunk. The TARP surgery, which included reimplantation of the whole great vessel block, was the primary choice for patients with extensive arch aneurysms or a sizable intimal lesion involving the inner arch. Patients with connective tissue disease or significant dislocation of

the great vessels underwent debranching and selective implantation. The same group of subjects were suitable recipients of the frozen elephant trunk (FET) option and received selective debranching/vessel implantation or insular reimplantation. The benefit provided by the 4-branch grafts was the ability to restore antegrade cardiopulmonary bypass through the reperfusion arm (lateral branch) of the graft utilized. Finally, to maintain systemic warming, a temperature gradient of 10°C between core blood and internal temperature was maintained during surgical hemostasis. During this phase, the residual suture line was strengthened following the previously described procedure. Once a body temperature of 36°C was reached, cardiopulmonary bypass was discontinued.

Table S1. Coefficients of the best model of LASSO logistic regression for early mortality.

**Variables**	**s0**
(Intercept)	-4.021
Age	0.0384
Gender	.
Weight	.
eGFR	-0.0158
Hemoglobin	.
Arterial lactate	0.1936
Family history of aortic dissection or aneurysm	-0.2424
Prior cardiac surgery	.
Hypertension	.
Diabetes	.
Stroke	.
Pulmonary disease	.
Extracardiac arteriopathy	.
Poor mobility	1.5932
Recent myocardial infarction	.
Left ventricular ejection fraction	
>50%	-0.1924
21-30%	.
31-50%	.
Systolic pulmonary artery pressure	.
Cardiogenic shock requiring inotropes	.
Cardiac tamponade	0.5621
Preoperative intubation	0.5180
Any malperfusion excluding myocardial malperfusion	0.2533
Tear in the aortic root	.
Tear in the ascending aorta	.
Tear in the aortic arch	.
Myocardial ischemic time	.
Cardiopulmonary bypass time	0.0023
Hypothermic circulatory arrest duration	.
Bicuspid aortic valve	0.1597
Aortic root replacement	.
CABG	.
Total or partial aortic arch repair	.
Antegrade cerebral perfusion	0.0498
Retrograde cerebral perfusion	-0.0529
Urgency of the procedure	
Emergency 1	.
Emergency 2	.
Salvage 1	0.0798
Salvage 2	.
Urgent	.

CABG, coronary artery bypass grafting; eGFR, estimated glomerular filtration rate. Best lambda value: 0.02001626.

Table S2. Accuracy of LASSO REGRESSION.

	True		
**Predicted**	0	1	Total
0	329	80	409
1	10	30	40
Total	339	110	449
Percent Correct: 0.7996			

Table S3. Variance inflation factor calculation for each independent variable of multiple logistic regression.

**Variable**	**VIF value**
Age	1.1350
eGFR	1.0973
Arterial lactate	1.0468
Family history of aortic dissection or aneurysm	1.0560
Poor mobility	1.0254
Cardiac tamponade	1.0311
Preoperative intubation	1.0312
Cardiopulmonary bypass time	1.0535

eGFR, estimated glomerular filtration rate; VIF, Variance inflation factor.

Best cut-off statistics

Method: maximize_spline_metric
Predictor: Arterial lactate
Outcome: Early mortality
Direction: >=
Nr. of bootstraps: 1000

AUC	n	n_pos	n_neg
0.6702	449	110	339

optimal_cutpoint	spline_sum_sens_spec	acc	sensitivity	specificity	tp	fn	fp	tn
2.6	1.2717	0.7283	0.4545	0.8171	50	60	62	277

**Predictor summary:**												
**Data**	Min.	5%	1st	Qu.	Median	Mean	3rd	Qu.	95%	Max.	SD	NAs
Overall	0.4	0.700	1.0	1.4	2.069.354	2.5	5.800	21	1.941.926	0		
0	0.4	0.700	0.9	1.2	1.741.947	2.2	4.600	8	1.272.211	0		
1	0.4	0.745	1.2	2.2	3.078.364	3.4	7.319	21	3.019.893	0		
Bootstrap summary:												
Variable	Min.	5%	1st	Qu.	Median	Mean	3rd	Qu.	95%	Max.	SD	NAs
optimal_cutpoint	1.10	2.00	2.40	2.60	2.61	2.80	3.20	5.00	0.39	0		
AUC_b	0.56	0.61	0.65	0.67	0.67	0.69	0.72	0.77	0.03	0		
AUC_oob	0.53	0.60	0.64	0.67	0.67	0.70	0.74	0.82	0.04	0		
sum_sens_spec_b	1.06	1.16	1.22	1.25	1.25	1.29	1.34	1.41	0.05	0		
sum_sens_spec_oob	0.98	1.11	1.19	1.24	1.24	1.29	1.36	1.47	0.08	0		
acc_b	0.51	0.66	0.70	0.72	0.72	0.74	0.77	0.80	0.03	0		
acc_oob	0.43	0.65	0.69	0.72	0.71	0.74	0.77	0.82	0.04	0		
sensitivity_b	0.20	0.32	0.40	0.44	0.45	0.49	0.59	0.81	0.08	0		
sensitivity_oob	0.07	0.26	0.36	0.43	0.43	0.50	0.60	0.77	0.10	0		
specificity_b	0.40	0.70	0.78	0.82	0.81	0.85	0.89	0.95	0.06	0		
specificity_oob	0.36	0.69	0.77	0.82	0.81	0.85	0.90	0.97	0.06	0		

cohens_kappa_b	0.05	0.16	0.22	0.25	0.25	0.29	0.33	0.40	0.05	0
cohens_kappa_oob	-0.02	0.11	0.19	0.24	0.23	0.28	0.35	0.44	0.07	0

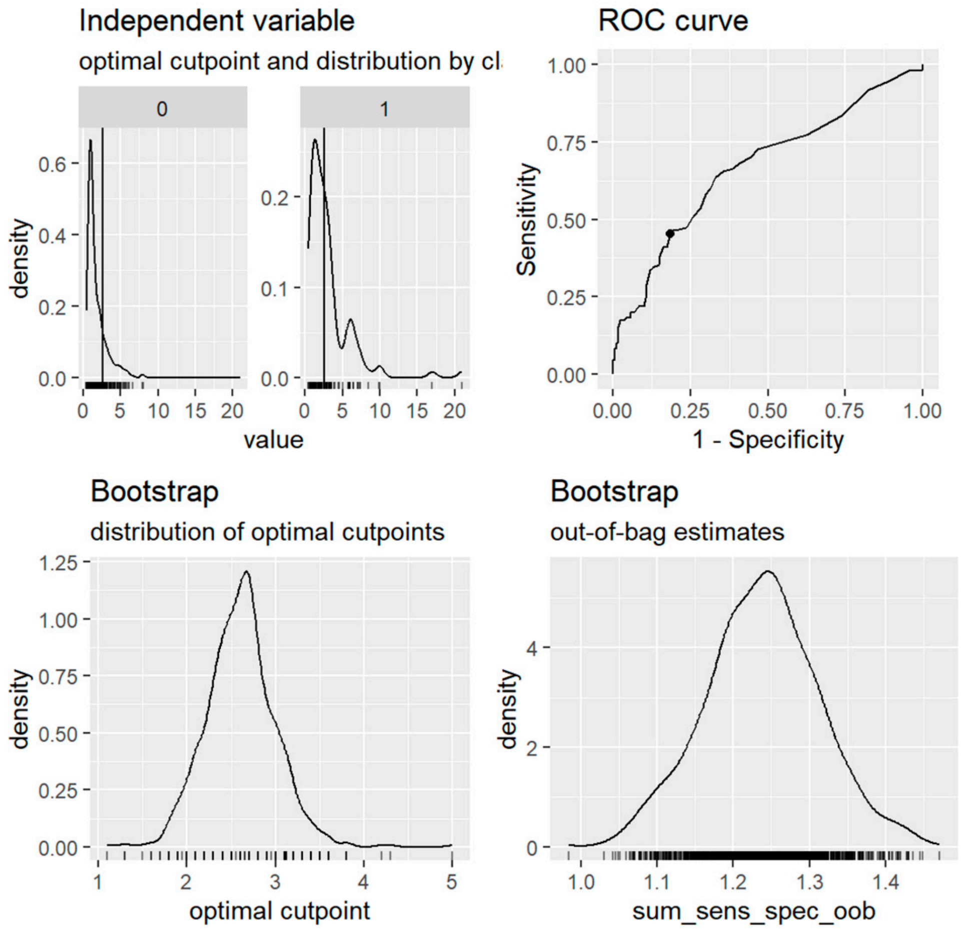

Figure S1. The top left plot shows predictor values per class, the top right shows the receiver operating characteristic curve (ROC curve), the bottom left showing the bootstrapped cutpoint variability, and the bottom right showing the distribution of the out-of-bag metric values (see best cut-off statistics in supplemental materials for details).
